# Comparison of the Glidescope^® ^and Pentax AWS^® ^laryngoscopes to the Macintosh laryngoscope for use by Advanced Paramedics in easy and simulated difficult intubation

**DOI:** 10.1186/1471-227X-9-9

**Published:** 2009-05-17

**Authors:** Sajid Nasim, Chrisen H Maharaj, Muhammad A Malik, John O' Donnell, Brendan D Higgins, John G Laffey

**Affiliations:** 1 Department of Anaesthesia, Galway University Hospitals, Galway, Ireland; 2 Department of Anaesthesia, Sligo General Hospital, Sligo, Ireland; 3 Department of Emergency Medicine, Galway University Hospitals, Galway, Ireland; 4 Department of Anaesthesia, Clinical Sciences Institute, National University of Ireland, Galway, Ireland

## Abstract

**Background:**

Intubation of the trachea in the pre-hospital setting may be lifesaving in severely ill and injured patients. However, tracheal intubation is frequently difficult to perform in this challenging environment, is associated with a lower success rate, and failed tracheal intubation constitutes an important cause of morbidity. Novel indirect laryngoscopes, such as the Glidescope^® ^and the AWS^® ^laryngoscopes may reduce this risk.

**Methods:**

We compared the efficacy of these devices to the Macintosh laryngoscope when used by 25 Advanced Paramedics proficient in direct laryngoscopy, in a randomized, controlled, manikin study. Following brief didactic instruction with the Glidescope^® ^and the AWS^® ^laryngoscopes, each participant took turns performing laryngoscopy and intubation with each device, in an easy intubation scenario and following placement of a hard cervical collar, in a SimMan^® ^manikin.

**Results:**

Both the Glidescope^® ^and the AWS^® ^performed better than the Macintosh, and demonstrate considerable promise in this context. The AWS^® ^had the least number of dental compressions in all three scenarios, and in the cervical spine immobilization scenario it required fewer maneuvers to optimize the view of the glottis.

**Conclusion:**

The Glidescope^® ^and AWS^® ^devices possess advantages over the conventional Macintosh laryngoscope when used by Advanced Paramedics in normal and simulated difficult intubation scenarios in this manikin study. Further studies are required to extend these findings to the clinical setting.

## Background

Intubation of the trachea by paramedics in the pre-hospital setting may be lifesaving in severely ill and injured patients [1-3]. However, tracheal intubation is often difficult to perform in this setting, is associated with a lower success rate. Failed tracheal intubation in this setting constitutes an important cause of morbidity, arising from direct airway trauma and the systemic complications of hypoxia [4,5]. In Ireland, Advanced Paramedics (AP's) are a subgroup of Emergency Medicine Technicians that are trained and certified as being competent in the skill of direct laryngoscopy and tracheal intubation.

**Figure 1:**
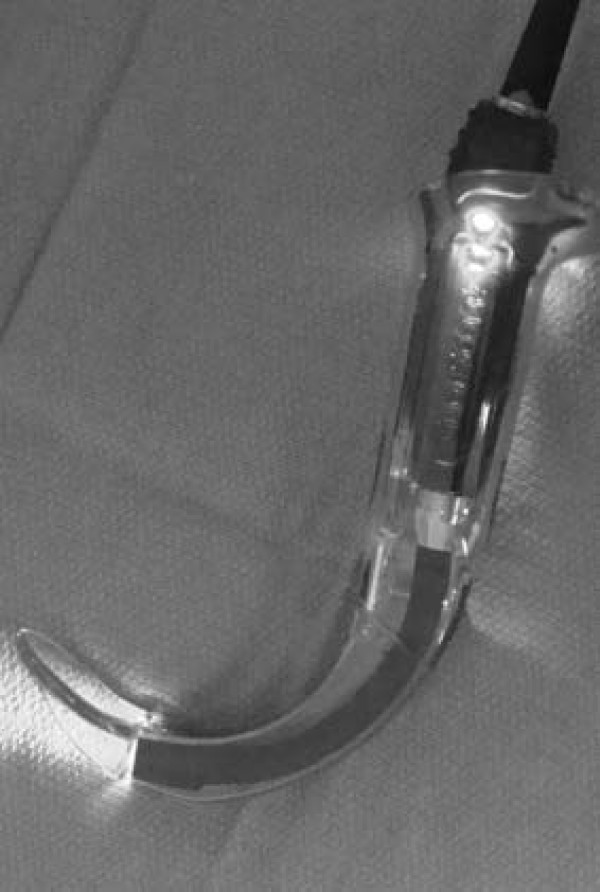
**Photograph of the Glidescope laryngoscope**. The device is held in the left hand and passed into the mouth over the tongue, and the tip placed in the vallecula or under the epiglottis.

**Figure 2:**
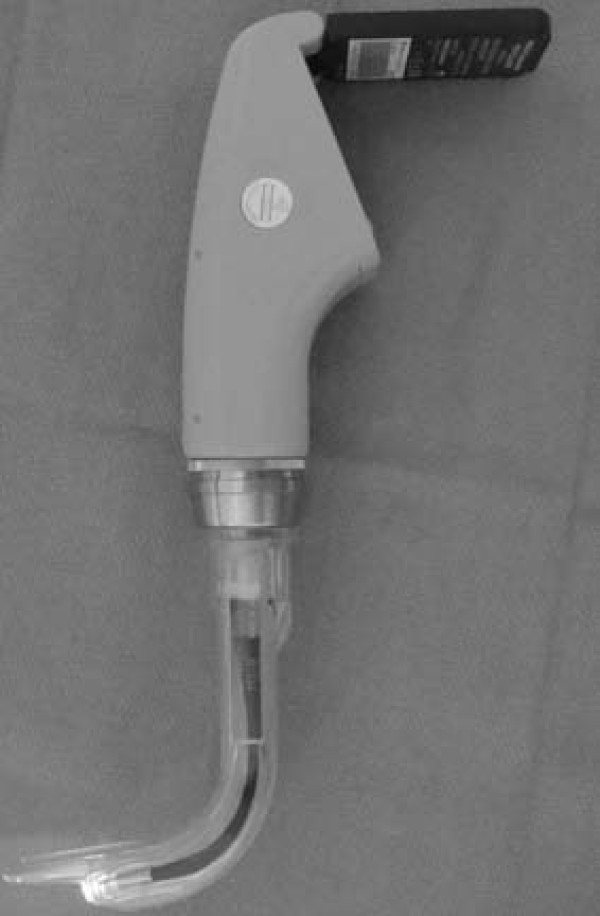
**Photograph of the AWS^® ^laryngoscope**. The device is held in the left hand and passed into the mouth over the tongue, and the tip is placed under the epiglottis.

The recent development of a number of indirect laryngoscopes, which do not require alignment of the oral-pharyngeal-tracheal axes, may reduce the difficult of tracheal intubation in the prehospital setting. Two portable indirect laryngoscopes, which could be included in ambulance equipment inventories, are the Glidescope^® ^(Saturn Biomedical System Inc., Burnaby, Canada) (Figure 1) and the AWS^® ^(Hoya Corporation, Tokyo, Japan) (Figure 2) laryngoscopes. Clinical studies have demonstrated advantages over the Macintosh laryngoscope for both the Glidescope^® ^[6-9], and the AWS^® ^[8,10] laryngoscopes. However, the efficacy of the Glidescope^® ^and the AWS^® ^when used by APs is not known, and the relative efficacies of these devices in comparison to the Macintosh laryngoscope have not been compared in a single study.

We therefore wished to compare these two novel devices to the Macintosh laryngoscope when used by APs with demonstrated competence in the skill of tracheal intubation using the Macintosh laryngoscope.

## Methods

Following ethical committee approval, and written informed consent, 25 Advanced Paramedics certified as competent to perform tracheal intubation consented to participate in this study. These participants constituted a convenience sample of AP's that attended a Resuscitation Conference on the 11^th ^June 2008 in Galway, Ireland, and represents more than 20% of all paramedics in Ireland.

Each AP received a standardized training session with the Glidescope^®^, the AWS^® ^and the Macintosh laryngoscopes. This included a demonstration of the intubation technique with each device, and verbal instructions regarding the correct use of each device. The use of optimization manoeuvres, such as external laryngeal pressure, to facilitate intubation with the Macintosh was also demonstrated. The total training time for each device was ten minutes. Each participant was then allowed to perform practice attempts with each device until each performed one successful tracheal intubation with each device. This training was carried out by a different member of the study team to the investigator that performed the actual study measurements. All intubations were performed with a 7.5 mm internal diameter cuffed endotracheal tube (ETT). The sequence in which each participant used the devices was initially randomized, and thereafter each participant used the devices in the same sequence throughout the protocol.

The design of the study was a randomized crossover trial. Each AP performed tracheal intubation with each device in a SimMan^® ^manikin (Laerdal^®^, Kent, UK) in the following laryngoscopy scenarios: (1) normal airway in the supine position; (2) cervical immobilization, achieved by mean of placement of a hard neck collar; and (3) normal airway in the supine position. The aim of the latter scenario was to determine whether there was a learning curve with the newer devices. The primary endpoints were the rate of successful placement of the endotracheal tube (ETT) and the duration of tracheal intubation. The duration of each tracheal intubation attempt was defined as the time taken from insertion of the blade between the teeth until the ETT was deemed to be correctly positioned by each participant. Where the participant visualized the ETT passing through the cords, the attempt was considered complete at this point. Where the participant was unsure as to the position of the ETT, the time taken to connect the ETT to an Ambu^® ^bag and inflate the lungs was also included in the duration of the attempt. In any case, after each intubation attempt an investigator verified the position of the ETT tip. A failed intubation attempt was defined as an attempt in which the trachea was not intubated, or where intubation of the trachea required greater than 60 seconds to perform [11-14].

Additional endpoints included the rate of successful placement of the endotracheal tube (ETT) in the trachea, the number of intubation attempts, the number of optimization maneuvers required (readjustment of head position, second assistant) to aid tracheal intubation and the severity of dental trauma. The severity of dental trauma was calculated based on a grading of pressure on the teeth (none = 0, mild = 1, moderate/severe ≥ 2). To improve reliability the same investigator assessed the severity of dental compression every time thus removing the potential for any inter-rater variability. We have demonstrated in multiple previous studies that this method of assessing dental pressure performs well, and appears to yield reasonably consistent results over time [15-20].

At the end of each scenario, each participant scored the ease of use of each device on a visual analogue scale (from 0 = Extremely Easy to 10 = Extremely Difficult). At the end of this protocol, each participant completed a questionnaire to determine self-assessed comfort and skill level for all three devices.

### Statistical analysis

We based our sample size estimation on the duration of the successful tracheal intubation attempt. Based on prior studies [15] we projected that the duration of tracheal intubation would be 15 seconds for the Macintosh laryngoscope, with a standard deviation of 5 seconds, in the easy laryngoscopy scenario with the Macintosh laryngoscope. We considered that an important change in the duration of tracheal intubation would be a 33% absolute change, i.e. an increase to 20 seconds or a reduction to 10 seconds. Based on these figures, using an α = 0.05 and β = 0.2, for an experimental design examining three devices, we estimated that 17 paramedics would be required. We therefore aimed to enroll a minimum of 20 paramedics to the study.

The analysis was performed using Sigmastat 3.5 (Systat Software, San Jose, CA, USA. Data for the duration of the first and the successful intubation attempt, the instrument difficulty score, and the overall device assessment were analyzed using one way Analysis of Variance (ANOVA) or the using the Kruskal-Wallis One Way ANOVA on Ranks depending on the data distribution. Data for the number of intubation attempts, number of optimization maneuvers, severity of dental trauma, and the instrument difficulty score were analyzed using the Kruskal-Wallis One Way Analysis of Variance on Ranks. Data for the success of tracheal intubation attempts was analyzed using Chi square test. Continuous data are presented as means ± standard deviation (SD) or median (interquartile range), and ordinal and categorical data are presented as number and as frequencies. The α level for all analyses was set as P < 0.05.

## Results

Twenty-five Advanced Paramedics were approached and each consented to participate in the study.

### Scenario 1 – Normal Airway Scenario

**Figure 3:**
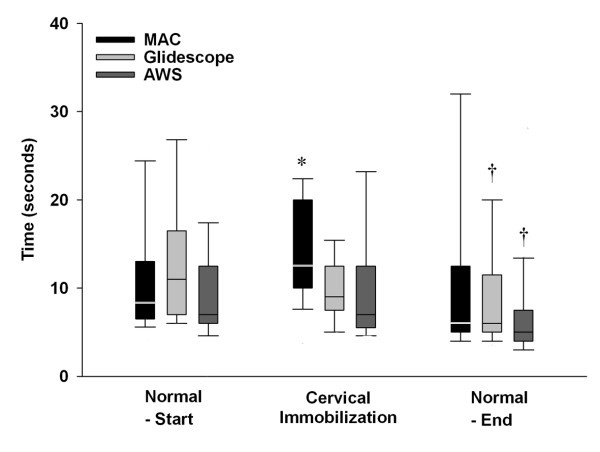
**Box plot representing the duration required to successfully intubate the trachea with each device in each scenario tested**. The data are given as median and interquartile range, with the bars representing the 10^th ^and 90^th ^centile. * Indicates significantly different compared to both other Laryngoscopes. **†**Indicates significantly different from same device at the start of the protocol. Labels: Normal – Start: Intubation of the normal airway at the start of the protocol; Cervical Immobilization – Immobilization of the neck with hard collar; Normal – End: Intubation of the normal airway at the end of the protocol.

All 25 APs successfully intubated the trachea with the Macintosh laryngoscope, the Glidescope^® ^and the AWS^® ^(Table 1). All 25 APs successfully intubated the trachea on the first attempt with the AWS^®^, while one AP needed a second attempt with the Macintosh and one with the Glidescope^® ^(Table 1). The duration of the first and of the successful tracheal intubation attempts, and the number of optimization maneuvers required with each device were not significantly different (Table 1 and Figure 3). The severity of dental compression was significantly greater with the Macintosh compared to both the Glidescope^® ^and AWS^® ^devices and was significantly greater with the Glidescope^® ^compared to the AWS^® ^device (Table 1). The participants found the AWS^® ^devices significantly easier to use than the Macintosh and Glidescope^® ^laryngoscopes in this scenario (Figure 3). There was no significant difference in difficulty of device use between the Macintosh and Glidescope^® ^laryngoscopes.

**Table 1:** Data from easy laryngoscopy scenario.

**Parameter Assessed**	**Macintosh**	**Glidescope**^®^	**AWS**^®^	**P value**
Overall Success Rate (%)	25 (100)	25 (100)	25 (100)	P = 1.0
				
Duration (sec, 1^st ^attempt)	8 (7, 13)	11 (7, 17)	7 (6, 12)	P = 0.1
				
No of Intubation Attempts (%)				
1	24 (96)	24 (96)	25 (100)	P = 0.602
2	1 (4)	1 (4)	0	
3	0	0	0	
				
No of Optimization Maneuvers				
0	23 (92)	25 (100)	25 (100)	P = 0.368
1	2 (8)	0	0	
> 1	0	0	0	
				
Dental Compression [Severity]				
0	1 (4)*	11 (44)*	18 (72)*	P < 0.001
Mild [+]	13 (52)	10 (40)	5 (20)	
Severe [++]	11 (44)	4 (16)	2 (8)	

**Notes: **Data are reported as mean ± SD, median (interquartile range) or as number (percentage).

* Significantly [P < 0.05] different compared to both other groups

### Scenario 2 – Cervical Spine Immobilization Scenario

**Figure 4:**
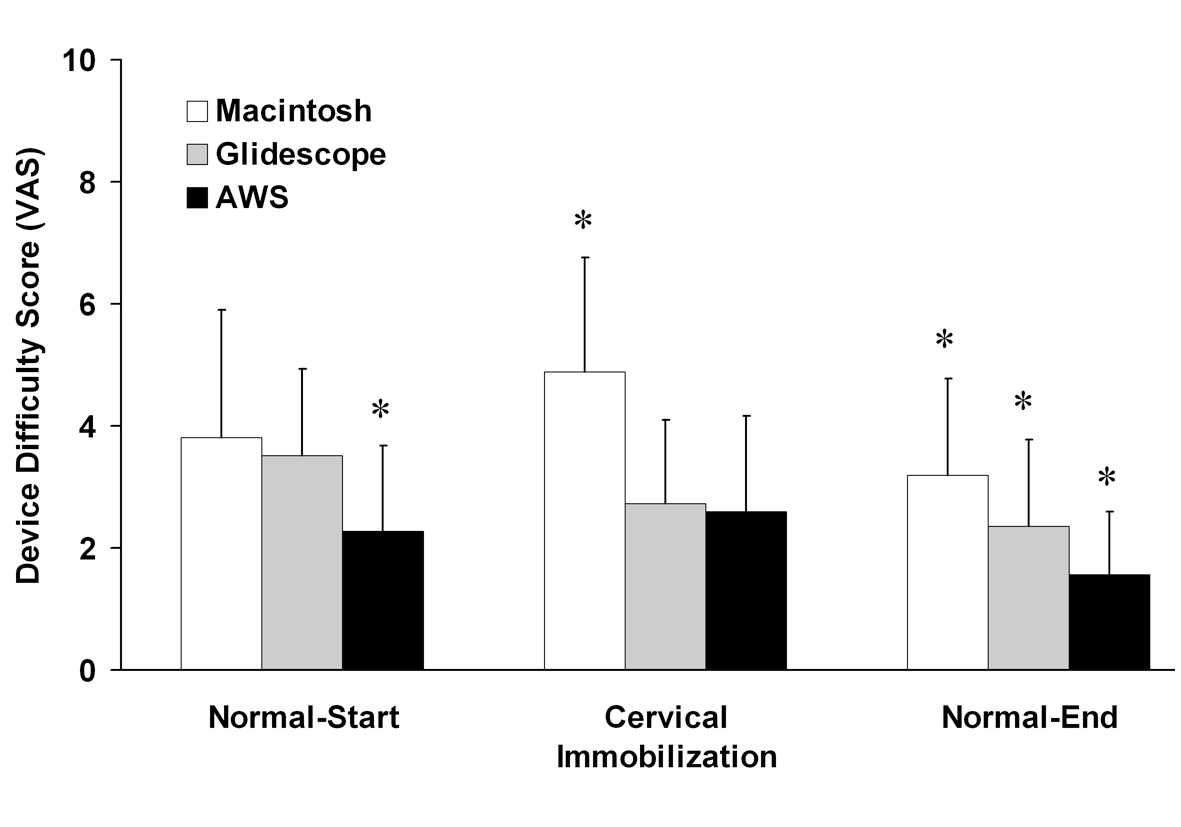
**Graph representing the user rated degree of difficulty of use of each instrument in each scenario tested**. The data are given as mean ± SD. * Indicates significantly different compared to both other Laryngoscopes. Labels: Normal – Start: Intubation of the normal airway at the start of the protocol; Cervical Immobilization – Immobilization of the neck with hard collar; Normal – End: Intubation of the normal airway at the end of the protocol.

All 25 APs successfully intubated the trachea with the Macintosh laryngoscope, the Glidescope^® ^and the AWS^® ^(Table 2). The duration of both the first and the successful tracheal intubation attempts were significantly longer with the Macintosh compared to the Glidescope^® ^and AWS^® ^devices (Table 2 and Figure 3). There was no significant difference in the duration of tracheal intubation attempts between the Glidescope^® ^and AWS^® ^devices (Table 2). There were no between group differences in the number of intubation attempts required with each device (Table 2). The number of optimization maneuvers required was significantly higher with the Macintosh compared to the Glidescope^® ^or AWS^® ^devices (Table 2). There was no difference in regard to the number of optimization maneuvers required with the Glidescope^® ^and AWS^® ^devices (Table 2). The severity of dental compression was significantly greater with the Macintosh compared to both the Glidescope^® ^and AWS^® ^devices (Table 2). There was no difference in severity of dental compression between the Glidescope^® ^and AWS^® ^devices (Table 2). The participants found the Macintosh laryngoscope significantly more difficult to use than the Glidescope^® ^or AWS^® ^devices in this scenario (Figure 4). There was no significant difference in difficulty of device use between the Glidescope^® ^and AWS^® ^laryngoscopes.

**Table 2:** Data from Cervical Immobilization scenario.

**Parameter Assessed**	**Macintosh**	**Glidescope**^®^	**AWS**^®^	**P value**
Overall Success Rate (%)	25 (100)	25 (100)	25 (100)	P = 1.0
				
Duration (sec, 1^st ^attempt)	13 (10, 20)*	9 (8, 12)	8 (6, 13)	P = 0.013
				
No of Intubation Attempts (%)				
1	23 (92)	25 (100)	25 (100)	P = 0.358
2	2 (8)	0	0	
3	0	0	0	
				
No of Optimization Maneuvers				
0	2 (8)*	24 (96)	25 (100)	P < 0.001
1	23 (92)	1 (4)	0	
> 1	0	0	0	
				
Dental Compression [Severity]				
0	0*	6 (24)	10 (40)	P = 0.023
Mild [+]	12 (48)	10 (40)	5 (20)	
Severe [++]	13 (52)	9 (36)	10 (40)	

**Notes: **Data are reported as mean ± SD, median (interquartile range) or as number (percentage).

* Significantly [P < 0.05] different compared to both other groups

### Scenario 3 – End protocol Normal Airway Scenario

All 25 APs successfully intubated the trachea on the first attempt with the Macintosh laryngoscope, and the Glidescope^®^, while one AP needed a second attempt with the AWS^® ^(Table 3). The duration of the first and of the successful tracheal intubation attempts, the number of intubation attempts, and the number of optimization maneuvers required with each device were not significantly different in this scenario (Table 3). The duration of tracheal intubation attempts was significantly shorter with the Glidescope^® ^and the AWS^® ^devices but not with the Macintosh laryngoscopes, in this scenario compared to the first scenario (Tables 1, 3 and Figure 3). The severity of dental compression was significantly greater with the Macintosh compared to both the Glidescope^® ^and AWS^® ^devices (Table 3). There was no difference in severity of dental compression between the Glidescope^® ^and AWS^® ^devices (Table 3). The participants found the Pentax^® ^AWS device significantly easier to use than the Macintosh and Glidescope^® ^laryngoscopes in this scenario. They also found the Glidescope^® ^laryngoscope significantly easier to use than the Macintosh laryngoscope (Figure 4).

**Table 3:** Data from repeat easy laryngoscopy scenario.

**Parameter Assessed**	**Macintosh**	**Glidescope**^®^	**AWS**^®^	**P value**
Overall Success Rate (%)	25 (100)	25 (100)	25 (100)	P = 1.0
				
Duration (sec, 1^st ^attempt)	6 (5, 12)	6 (5, 11)	5 (4, 8)	P = 0.25
				
No of Intubation Attempts (%)				
1	25 (100)	25 (100)	24 (96)	P = 0.368
2	0	0	1 (4)	
3	0	0	0	
				
No of Optimization Maneuvers				
0	24 (96)	25 (100)	25 (100)	P = 0.368
1	0	0	0	
> 1	1 (4)	0	0	
				
Dental Compression [Severity]				
0	4 (16)*	15 (60)	20 (80)	P < 0.001
Mild [+]	11 (44)	4 (16)	2 (8)	
Severe [++]	10 (40)	6 (24)	3 (12)	

**Notes: **Data are reported as mean ± SD, median (interquartile range) or as number (percentage).

* Significantly [P < 0.05] different compared to both other groups

### End protocol overall device assessment

The APs found the Macintosh significantly more difficult to use than the Glidescope^® ^and AWS^® ^devices (Table 4). There was no significant difference in the ease of use of the Glidescope^® ^and AWS^® ^devices (Table 4). The APs expressed similar levels of confidence in performing tracheal intubation with each of the devices tested (Table 4).

**Table 4:** Overall Device assessment by Participants.

**Parameter Assessed**	**Macintosh**	**Glidescope^®^**	**AWS^®^**	**P value**
Overall Difficulty of Use Score	4.3 ± 2.0*	2.9 ± 2.1	2.6 ± 2.1	P = 0.01
				
Overall Confidence with each device	7.4 ± 1.5	7.1 ± 2.4	7.8 ± 2.6	P = 0.52

**Notes: **Data are reported as mean ± SD or as number (percentage).

* Significantly [P < 0.05] different compared to both other groups

## Discussion

In Ireland, Advanced Paramedics are trained and certified as being competent in the skill of direct laryngoscopy and tracheal intubation by the Pre-Hospital Emergency Care Council (PHECC). Following initial training on high fidelity manikins, each AP is then seconded to a hospital for clinical training in the operating suite. Each AP must perform a minimum of 10 successful tracheal intubations under the direct supervision of a senior anaesthetist prior to certification. Once in clinical practice, AP's perform an average of 10–12 tracheal intubations per person per year. Consequently, this cohort possesses a high level of competence in the skill of tracheal intubation, and maintains this skill once in practice.

Outcome in severely ill and injured patients is improved where the airway is successfully secured early by tracheal intubation [1-3]. However, where difficulties or complications arise as a results of difficulties or failure to secure the airway in the pre-hospital patient, significant morbidity and even mortality may ensue [4,5,21]. The pre-hospital environment is a challenging one, and tracheal intubation is frequently difficult to perform and associated with a lower success rate compared to the hospital setting [22]. The need for repeated attempts to secure the airway emergently increases airway-related complications such as hypoxia, pulmonary aspiration and adverse hemodynamic events [5]. Accidental esophageal intubation can result in catastrophic complications, including pulmonary aspiration of gastric contents, cerebral hypoxia, and cardiac arrest [4]. Difficulties in tracheal intubation may also result in severe local complications such as perforation of laryngeal or pharyngeal structures [23].

Given these issues, the practice of pre-hospital tracheal intubation by personnel not fluent in the technique is increasingly questioned [24-26]. A slow learning curve for intubation with the Macintosh blade has been well documented among paramedic personnel [27,28] due to lack of regular exposure to the technique. These difficulties have led to the increasing use of alternative airway devices such as Combitube^®^, Laryngeal Tube^® ^and Laryngeal Mask Airway^® ^for airway management in the pre-hospital setting contexts [29-31], due to the rapid learning curves associated with these devices [32,33]. However trauma to the airway and aspiration injury remains a significant risk with these devices in these patients.

Conventional direct laryngoscopic laryngoscopes, such as the Macintosh laryngoscope, require the alignment of oral and tracheal axes in order to view the glottic opening. This is a difficult skill to successfully acquire [26,27,34], and to maintain [28], particularly if the opportunities to practice this skill are limited. Both the Glidescope^® ^(Figure 1) and AWS^® ^(Figure 2) devices have an exaggerated blade curvature with enhanced optics that give a view of the glottis without the need to align the oral and tracheal axes. Both devices are portable, and could be easily included in ambulance equipment inventories. We therefore wished to determine whether these devices possessed advantages over the conventional Macintosh laryngoscope when used by paramedics in the setting of normal and simulated difficult intubation.

Our study demonstrated that both the Glidescope^® ^and AWS^® ^devices demonstrated several advantages over the Macintosh laryngoscope, in both the normal and in the difficult intubation scenario. Both devices reduced the duration of tracheal intubation attempts in the cervical immobilization scenario, a situation commonly seen in the emergency pre-hospital setting. While the degree to which these devices reduced the time required to secure the airway appears relatively small, of the order of 5 – 10 seconds, it must be remembered that brain hypoxia may rapidly supervene in the emergency setting. In addition, these devices reduced the number of optimization maneuvers and reduced the potential for dental trauma when compared to the Macintosh laryngoscope. Of the two indirect laryngoscopes studies, despite largely comparable performance in other measures of difficulty, the APs found the AWS^® ^easier to use in each scenario. The AWS^® ^caused the least amount of dental compressions in each scenario. The structure of the blade of the AWS^®^, particularly the incorporation of a side channel for the ETT, may explain its better performance in these respects compared to the Glidescope.

Both the Glidescope^® ^and AWS^® ^devices exhibited a rapid learning curve, despite a deliberately brief instruction period. In the repeated easy laryngocsopy scenario, the duration of intubation attempts were significantly reduced for both the Glidescope^® ^and AWS^® ^laryngoscopes compared to the first scenario. This finding supports the feasibility of introducing these devices for use by APs in the out-of-hospital setting, and adds to the growing body of literature attesting to the fact that these devices, by reducing the skill required to perform tracheal intubation, may be particularly for advanced life support personnel, that may be called upon to perform this skill relatively infrequently [35-38]. In their overall assessment of the devices studied, the AP's rated the Macintosh most difficult device to use in each scenario. However, when assessing their confidence in the use of each device for tracheal intubation procedures, they rated the three laryngoscopes similarly. This rating probably reflects the familiarity of the AP's with the Macintosh laryngoscope.

A number of important limitations exist regarding this study. Firstly, we acknowledge that the potential for bias exists, as it is impossible to blind the AP's to the device being used. Secondly, this study was carried out in experienced users of the Macintosh laryngoscope. The findings may differ if studies in APs prior to their attaining competence with the Macintosh device. Thirdly, this is a manikin study, and may not adequately mimic clinical conditions, particularly in the emergency setting. A particular issue of particular relevance in the emergency setting is the risk of fogging and contamination of the lens by secretions and/or blood, especially in the traumatized airway. Therefore, these findings need to be confirmed and extended in clinical studies before definitive conclusions can be drawn. Careful consideration would also have to be given to the cost implications of introducing these more expensive laryngoscopes into the pre-hospital emergency care setting. Finally, the relative efficacies of these devices in comparison to other promising devices such as the Airtraq^® ^[39,40], McCoy^® ^[41], McGrath^®^[19], TruView^® ^[42], LMA CTrach^® ^[14] or Bonfils^® ^[43] have not been determined. We focused on the Glidescope^® ^and AWS^® ^devices in this study due to the fact that these are portable devices that can easily be included in the equipment used by AP's. Nevertheless, further comparative studies are needed with other alternative laryngoscopy devices in order to find the optimal alternatives to the Macintosh laryngoscope.

## Conclusion

The Glidescope^® ^and AWS^® ^devices appears to possess advantages over the conventional Macintosh laryngoscope when used by AP's in normal and simulated difficult intubation scenarios. The AWS^® ^performed best overall, and demonstrates considerable promise in this context. Further clinical studies are necessary to confirm these initial positive findings.

## Abbreviations

ANOVA: analysis of variance; ETT: endotracheal tube; SD: standard deviation; VAS: Visual analogue scale.

## Competing interests

Pentax Ltd provided the AWS^® ^device and the disposable blades free of charge for this study.

## Authors' contributions

SN and CM conceived of the study, and participated in its design and execution and helped to draft the manuscript. AM, JO'D, and BDH participated in the study, recruited participants, and helped to draft the manuscript. JL participated in the design and coordination of the study, performed the statistical analysis, and helped to draft the manuscript. All authors read and approved the final manuscript.

## Pre-publication history

The pre-publication history for this paper can be accessed here:


